# [^18^F]FDG PET/CT-Derived Metabolic Characteristics and Tumour-Infiltrating Lymphocytes for Predicting Axillary Response to Neoadjuvant Chemo-(Immuno)Therapy in Clinically Node-Positive Breast Cancer

**DOI:** 10.3390/cancers18203302

**Published:** 2026-10-14

**Authors:** Melissa Lenaerts, Loes F. S. Kooreman, Chris C. P. Adang, Florien J. G. van Amstel, Sander M. J. van Kuijk, Paul J. van Diest, Felix M. Mottaghy, Cristina Mitea, Ernest J. T. Luiten, Carmen C. van der Pol, Linetta B. Koppert, Janine M. Simons, Marjolein L. Smidt, Thiemo J. A. van Nijnatten, on behalf of the REFINE Study Group

**Affiliations:** 1 Department of Surgery, Maastricht University Medical Center+, 6229 HX Maastricht, The Netherlands; 2 GROW—Research Institute for Oncology and Reproduction, Maastricht University, 6229 HX Maastricht, The Netherlands; 3 Department of Pathology, Maastricht University Medical Center+, 6229 HX Maastricht, The Netherlands; 4 Department of Radiology and Nuclear Medicine, Maastricht University Medical Center+, 6229 HX Maastricht, The Netherlands; 5 Department of Clinical Epidemiology and Medical Technology Assessment, Maastricht University Medical Center+, 6229 HX Maastricht, The Netherlands; 6 Department of Pathology, University Medical Center Utrecht, 3584 CX Utrecht, The Netherlands; 7 Department of Nuclear Medicine, RWTH Aachen University Hospital, 52074 Aachen, Germany; 8 Center of Integrated Oncology Aachen Bonn Cologne Duesseldorf (CIO ABCD), 52074 Aachen, Germany; 9 College of Medicine and Health Sciences, UAE University, Al Ain P.O. Box 17666, United Arab Emirates; 10 Department of Surgical Oncology, Alrijne Hospital, 2353 GA Leiderdorp, The Netherlands; 11 Department of Surgical Oncology, Erasmus Medical Center, 3015 GD Rotterdam, The Netherlands; 12 Department of Radiotherapy, Erasmus MC Cancer Institute, University Medical Centre Rotterdam, 3015 GD Rotterdam, The Netherlands; 13 Dutch Expert Center for Screening (LRCB), 6538 SW Nijmegen, The Netherlands

**Keywords:** breast neoplasms, lymph nodes, neoadjuvant therapy, positron emission tomography computed tomography, tumour-infiltrating lymphocytes

## Abstract

**Background**: Accurate axillary response prediction is clinically relevant in clinically node-positive (cN+) breast cancer patients treated with neoadjuvant chemo-(immuno)therapy (NAC). This study evaluated the value of [^18^F]FDG PET/CT-derived metabolic characteristics and tumour-infiltrating lymphocytes (TILs) to predict axillary pathologic complete response (pCR). Second, the association between [^18^F]FDG PET/CT-derived metabolic characteristics and TILs was evaluated. **Methods**: This retrospective study included cN+ breast cancer patients treated with NAC and axillary surgery from the prospective multicentre RISAS trial (NCT02800317). Baseline [^18^F]FDG PET/CT-derived metabolic activity of the breast tumour, axillary lymph node metastases (ALNMs), and spleen was assessed, together with TILs measured in the breast tumour and ALNMs. TILs were classified as low (<10%) versus high (≥10%). Associations were evaluated using multivariable logistic regression and receiver operating characteristic analysis. **Results**: Of 135 patients, 46 (34.1%) achieved axillary pCR. Higher liver mean tumour-to-background ratio (2.7 vs. 1.9, *p* = 0.042), trends towards higher breast and ALNM maximum standardised uptake value (SUVmax), lower splenic SUVmax and high breast tumour TILs were associated with axillary pCR. Multivariable logistic regression combining tumour grade, subtype, maximum spleen-to-liver ratio, and breast tumour TILs yielded an apparent area under the curve of 0.80 [95%CI 0.73–0.88]. Higher ALNM SUVmax and SUVmean (10.5 vs. 6.3, *p* = 0.024; 6.4 vs. 3.7, *p* = 0.015) and lower splenic SUVmean (2.6 vs. 3.1, *p* = 0.035) were associated with high breast tumour TILs. **Conclusions**: A model combining clinicopathological, metabolic, and immune-related characteristics may be informative for predicting axillary response to NAC but did not perform better than clinicopathological characteristics alone. Future studies should evaluate whether accurate prediction models can support safe axillary treatment de-escalation.

## Simple Summary

Breast cancer patients whose disease has spread to axillary lymph nodes usually receive chemotherapy before surgery. In some patients, this treatment fully clears the cancer from the axillary lymph nodes, meaning that removing them surgically may not be necessary. However, currently it is not yet possible to reliably identify these patients prior to surgery. This study examined whether measurements from [^18^F]FDG PET/CT at diagnosis, combined with the amount of tumour-infiltrating lymphocytes, could help predict which patients would have their axillary lymph nodes cleared of cancer after chemotherapy. Among 135 patients, a model combining tumour features, [^18^F]FDG PET/CT measurements, and tumour-infiltrating lymphocytes information predicted this outcome with good accuracy. These findings suggest that such a model could eventually help identify patients who may safely avoid axillary lymph node surgery; however, this needs to be confirmed in future research.

## 1. Introduction

In recent years, patients with clinically node-positive (cN+) breast cancer are more frequently treated with neoadjuvant chemo-(immunotherapy) (NAC) [1]. In about one third of cN+ breast cancer patients, NAC results in a pathologic complete response (pCR) of axillary lymph node metastases (ALNMs) [2,3]. Achieving axillary pCR may have important therapeutic implications, potentially de-escalating axillary surgery and adjuvant treatment.

In current clinical practice, less invasive axillary surgical staging procedures, such as sentinel lymph node biopsy (SLNB), marking axillary lymph nodes with an iodine seed (MARI) or targeted axillary dissection (TAD), are preferred over axillary lymph node dissection (ALND) after NAC [2,4,5]. The goal of axillary surgical staging is to identify axillary pCR after NAC in cN+ breast cancer patients. However, accurate response prediction before and during NAC may allow the identification of selected patients who will achieve axillary pCR, for whom axillary surgical staging could therefore be safely omitted.

Previous studies have tried to predict the response of breast tumours and ALNMs to NAC using imaging modalities and histopathological information. [^18^F]fluorodeoxyglucose ([^18^F]FDG) positron emission tomography/computed tomography (PET/CT) imaging can be considered in cN+ breast cancer for distant staging at diagnosis, as recommended by guidelines of the NCCN, EANM, and SNMMI [6,7]. [^18^F]FDG PET/CT provides insight into the metabolic activity of physiological and pathological cells [8]. Previous studies found that metabolic activity measured in tumour tissue may serve as an indirect marker of proliferation and oncological aggressiveness, providing predictive information regarding axillary pCR within breast cancer subtypes [9,10,11]. Alongside evaluating [^18^F]FDG uptake in tumour cells, immune-related organs such as the spleen are increasingly being investigated on [^18^F]FDG PET/CT as an indirect marker of systemic immune activity against tumour tissue. Multiple studies have demonstrated high splenic metabolic activity as a predictor of shorter survival in breast cancer and other cancer types [12,13,14,15]. In addition, tumour-infiltrating lymphocytes (TILs) are frequently used as a pathological marker that measures the immune response to tumour cells and serves as an independent predictor of response to NAC in breast cancer patients [16,17]. Previous studies have reported increased levels of breast tumour TILs to be correlated with an enhanced likelihood of pCR after NAC and a better recurrence-free survival, particularly in human epidermal growth factor receptor 2 (HER2)-positive and triple negative (TN) breast cancer subtypes [16,17,18]. However, while breast tumour TILs have been extensively studied, the assessment of pre-NAC ALNM TILs remains rarely performed in breast cancer, and their role in response prediction is still unknown.

Only a few studies have investigated the correlation between metabolic characteristics derived from [^18^F]FDG PET/CT and the level of TILs [19,20,21,22]. In general, these studies found weak-to-moderate correlations, with higher breast tumour metabolic activity corresponding with higher levels of breast tumour TILs, particularly in patients with HER2-positive and TN breast cancer [19,21,22]. In addition, the interplay between the systemic immune response, reflected by splenic metabolic activity, and the tumour-associated immune response, reflected by TILs, has been explored in only a few studies. Tamam et al. found a positive correlation between splenic metabolic activity and breast tumour TILs in stage 4 nonluminal breast cancer patients [20]. In contrast, Seban et al. observed an inverse association, demonstrating that a higher spleen-to-liver ratio (SLR) was associated with lower breast tumour TILs [15]. However, the combined potential of [^18^F]FDG PET/CT-derived metabolic characteristics and TILs to predict axillary pathological response to NAC has yet to be investigated.

Therefore, this study aimed to evaluate the value of baseline [^18^F]FDG PET/CT-derived metabolic activity of the breast tumour, ALNMs, and the spleen, as well as levels of pre-NAC TILs measured in the breast tumour and ALNMs, to predict axillary pathologic response in cN+ breast cancer patients treated with NAC overall and according to breast cancer subtype. Second, the association between [^18^F]FDG PET/CT-derived metabolic characteristics and TILs was evaluated.

## 2. Methods

### 2.1. Patients

This study presents a retrospective analysis of data collected prospectively in the multicentre RISAS trial (NCT02800317; Registration date: 15 June 2016; METC number 2016-412). Participants were women (≥18 years old) diagnosed with cN+ breast cancer and treated with NAC. NAC was administered according to national guidelines and generally consisted of anthracycline and/or taxanes. HER2-positive breast cancer patients received HER2-targeted therapy, consisting of trastuzumab with or without pertuzumab. Afterwards, surgery of the breast (except in patients with occult breast cancer) and ALNMs was performed, consisting of breast-conserving surgery or mastectomy combined with the RISAS procedure (SLNB + MARI) followed by completion ALND (cALND). Patients were excluded if they had infraclavicular or supraclavicular lymph node metastases (cN3a/cN3c), (oligo)metastatic breast cancer, SLNB performed before NAC or a history of radiotherapy to the ipsilateral axilla. Participants were recruited from 13 centres across the Netherlands between 1 March 2017 and 31 December 2019. Informed consent was obtained from all participants. Additional details regarding the RISAS trial have been previously published [5,23].

**Figure 1:**
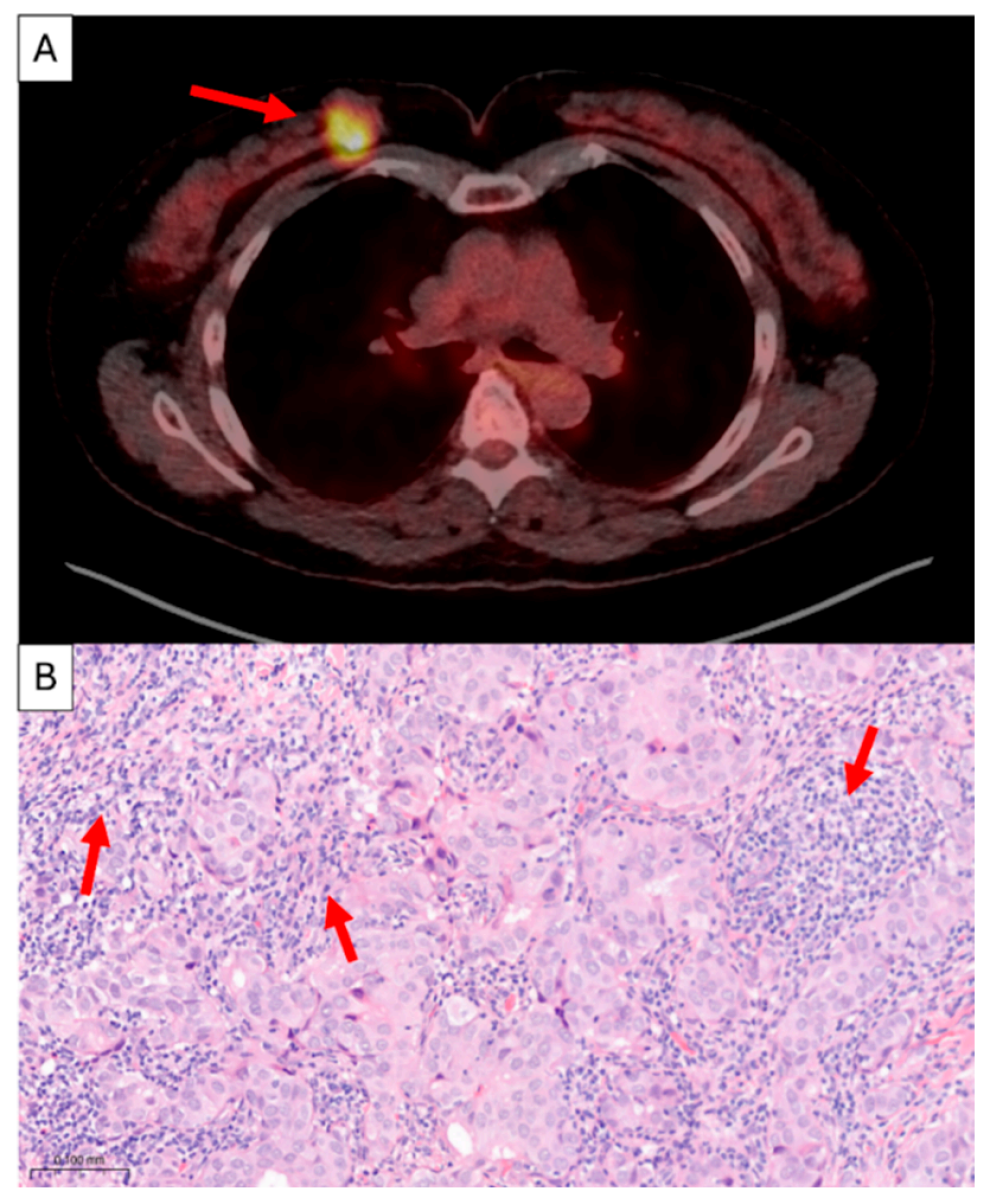
A 61-year-old patient diagnosed with HR-positive/HER2-negative grade 2 breast cancer. (**A**) [^18^F]FDG PET/CT exam showing high metabolic activity of the breast tumour (SUVmax = 14.07; red arrow). (**B**) Core needle biopsy of the breast tumour demonstrating high TILs (90%; red arrows). HR, hormone receptor; HER2, human epidermal growth factor receptor 2; SUVmax, maximum standardised uptake value; TILs, tumour-infiltrating lymphocytes.

This retrospective study included all RISAS participants who underwent baseline whole-body [^18^F]FDG PET/CT imaging and had a core needle biopsy (CNB) of the breast tumour and/or CNB of ALNMs. Fine-needle aspiration cytology specimens of ALNMs were excluded. Breast pCR was defined as the absence of any residual invasive or in situ disease in the breast (ypT0). Axillary pCR was defined as the absence of any residual disease in the axilla, including the absence of isolated tumour cells (ypN0). [^18^F]FDG PET/CT imaging and TILs were centrally evaluated specifically for the purpose of this retrospective study (Figure 1).

### 2.2. [^18^F]FDG PET/CT Imaging

All included patients underwent whole-body [^18^F]FDG PET/CT imaging in supine position before the start of NAC, using a standard acquisition protocol. Blood glucose levels had to be <11 mmol/L after a fasting period of at least 4–6 h before injection of [^18^F]FDG. Following blood glucose assessment, [^18^F]FDG was administered intravenously to all patients. [^18^F]FDG PET/CT imaging was performed after a resting period of 45–60 min. All included [^18^F]FDG PET/CT exams were reconstructed according to European Association of Nuclear Medicine Research Ltd. (EARL) guidelines [6,24,25].

[^18^F]FDG PET/CT images were reviewed by a dedicated nuclear radiologist (T.v.N) with 6 years of clinical experience using a commercially available workstation (syngo.via 10; Siemens Healthcare, Erlangen, Germany). The maximum standardised uptake value (SUVmax) and SUVmean of the breast tumour, most hypermetabolic ALN, contralateral breast, mediastinal blood pool, liver, and spleen were measured. In addition, the metabolic tumour volume (MTV) of the breast tumour was measured. In case of multifocality of the breast tumour, the SUVmax, SUVmean, and MTV of the most hypermetabolic lesion were assessed. The number of hypermetabolic ALNs was assessed by the reader (T.v.N.) and then compared with the number of ALNs reported by the initial institution. When the number of hypermetabolic ALNs was missing from the original report or when there was a difference between the number of hypermetabolic ALNs in the original report and the first reviewer, a second reviewer (C.M.) evaluated the images to obtain consensus. The nodal-to-tumour ratio (NT-ratio) was calculated by dividing the SUVmax or SUVmean of the most hypermetabolic ALN by the SUVmax or SUVmean of the breast tumour. Liver SUVmax and SUVmean values were obtained by placing a 30 mm diameter volume of interest (VOI) in the right lobe of the liver while avoiding abnormalities such as cysts, haemangiomas, or other structural lesions. For the contralateral breast measurement, a VOI was placed in evidently normal breast tissue on the side opposite to the tumour, positioned as symmetrically as possible relative to the affected breast. In case of bilateral breast cancer, a region clearly free of tumour involvement was selected. For the mediastinal blood pool measurement, a VOI was positioned in the ascending aorta just proximal to the aortic arch, avoiding the aortic wall to prevent measurement bias. The tumour-to-background ratio (TBR) was calculated by dividing the SUVmax or SUVmean of the breast tumour by the SUVmax or SUVmean of the background tissue (liver, contralateral breast, or mediastinal blood pool). Splenic SUVmax and SUVmean values were measured by placing a 20 mm diameter spherical VOI in the central part of the spleen on the axial slice showing the largest cross-section of the spleen [26,27]. The SLR was calculated by dividing the SUVmax or SUVmean of the spleen by the SUVmax or SUVmean of the liver.

### 2.3. Pathologic Evaluation

All available Haematoxylin and Eosin (H&E)-stained tissue sections of the breast and ALNM CNBs were centrally collected (PALGA 2022-109-A1-6) and digitised (Pannoramic p1000 scanner, 3DHistech, Budapest, Hungary). The percentage of stromal TILs was determined on all available breast tumour and ALNM CNBs. TILs were scored according to the International TILs working group guideline by an experienced breast pathologist with 12 years of experience in breast pathology (L.K.), blinded to additional clinical information [28]. Reporting TILs in ALNMs is challenging, because leukocytes are part of the normal lymph node structure. Only the lymphocytes in altered stroma surrounding the tumour cells were registered [19,28]. TILs were later classified as low (<10%) versus high (≥10%) [29,30]. All evaluations were performed using SlideViewer (Version 2.6, 3DHistech, Budapest, Hungary).

### 2.4. Statistical Analysis

Statistical analysis of the data was performed using the Statistical Package for the Social Sciences (Version 28, IBM Corp., Armonk, NY, USA). Continuous variables were described as mean ± standard deviation (SD) or median (range) and compared using the unpaired t-test or Mann–Whitney U test. Categorical variables were described as absolute numbers and percentages and compared using Pearson’s Chi-square test. Univariable and multivariable logistic regression analyses were performed to determine factors that influence axillary pCR. Associations were expressed as odds ratios (ORs) with 95% confidence intervals (CI) and *p*-values. Splenic metabolic activity and TILs were included in the multivariable logistic regression model, given their predefined clinical relevance and the aim of the study. Additional clinicopathological variables showing a significant association in univariable regression (*p* < 0.05) were selected for the multivariable logistic regression model, while taking into account recommendations regarding the number of events per variable (EPV) to limit model overfitting [31,32]. Receiver operating characteristic (ROC) curves were constructed, and areas under the curve (AUC) were computed to see how accurately the combined variables could discriminate between axillary pCR and axillary residual disease. Pre-NAC SLRmax and breast tumour TIL levels were compared between patients with and without axillary pCR within each breast cancer subtype using the Mann–Whitney U test. All statistical tests were two-sided, and a *p*-value < 0.05 was considered statistically significant.

## 3. Results

**Figure 2:**
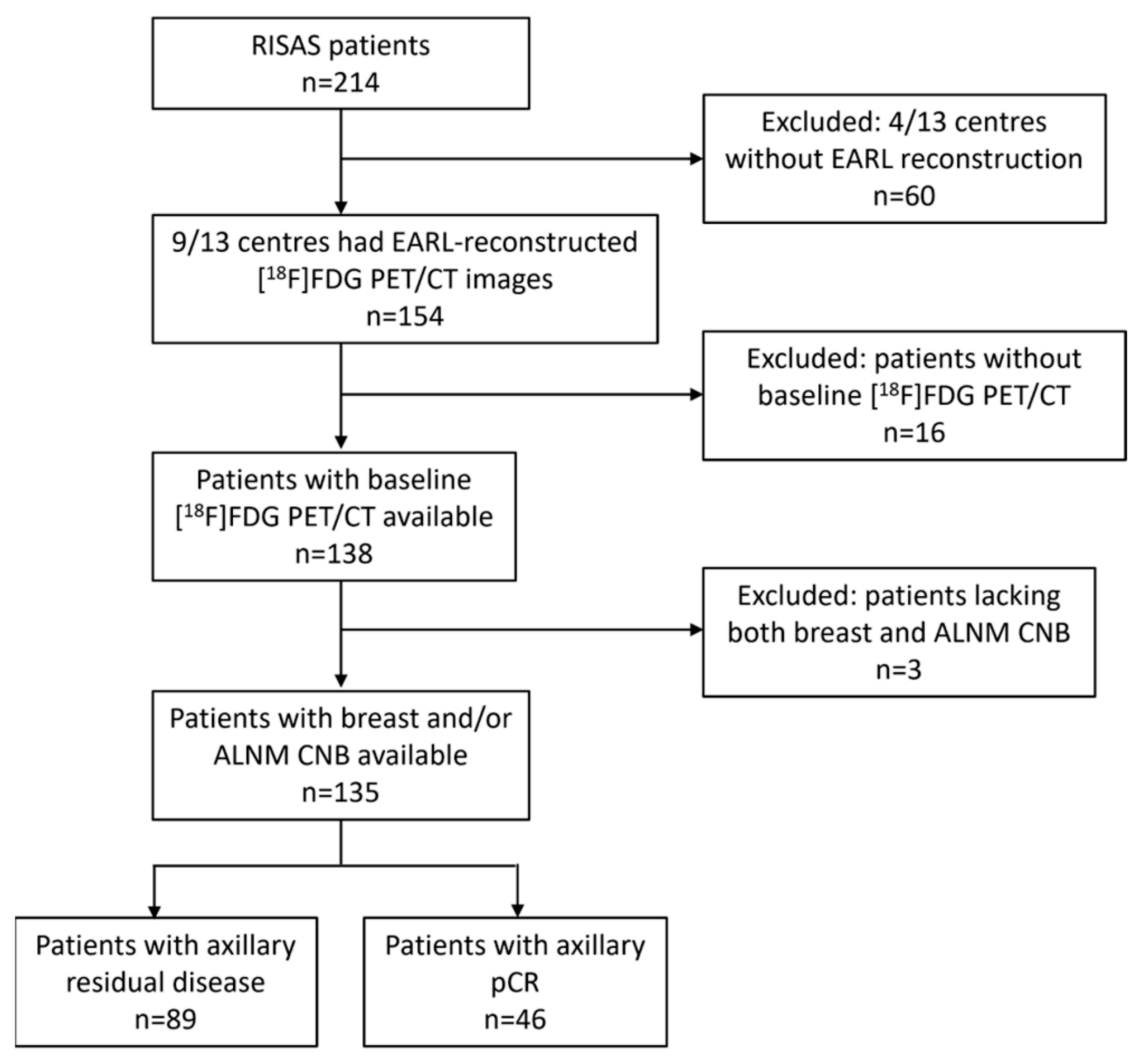
Flowchart showing patient selection process. RISAS, radioactive iodine seed placement in the axilla with sentinel lymph node biopsy; EARL, European Association of Nuclear Medicine Research Ltd.; ALNM, axillary lymph node metastases; CNB, core needle biopsy; pCR, pathological complete response.

A total of 214 patients were included in the RISAS trial. After excluding patients from centres without EARL-reconstructed [^18^F]FDG PET/CT exams, patients without a baseline [^18^F]FDG PET/CT exam, and patients lacking both breast tumour and ALNM CNB for TIL assessment, 135 patients were included in this retrospective analysis (Figure 2). All patients underwent surgery of ALNMs after NAC. The overall breast and axillary pCR rates were 33.6% (45/134) and 34.1% (46/135), respectively. A total of 29.1% (39/134) achieved combined pCR of both breast and axilla. Patient and tumour characteristics, according to axillary pCR status, are presented in Table 1. Axillary pCR was significantly more often achieved in patients with oestrogen receptor (ER)-negative, progesterone receptor (PR)-negative, HER2-positive, and grade 3 tumours.

Table 2 presents the [^18^F]FDG PET/CT imaging characteristics according to axillary pCR status. Among all evaluated characteristics, only TBRmean of the liver was significantly higher in patients who achieved axillary pCR (2.7 (range 0.2–6.7) vs. 1.9 (range 0.3–6.3), *p* = 0.042). For the remaining breast tumour and ALNM metabolic characteristics, only non-significant trends were observed, suggesting higher metabolic activity in patients who achieved axillary pCR. However, upon stratification by breast cancer subtype, this pattern shifted, with a trend towards lower breast tumour SUVmax values being associated with axillary pCR in all subtypes except TN breast cancer (Online Resource 1). Additionally, although not statistically significant, both splenic SUVmax and SLR demonstrated a trend towards lower metabolic activity in patients who achieved axillary pCR (Table 2 and Online Resource 1).

High breast tumour TILs (≥10%) were more frequently observed in patients with axillary pCR than in patients with axillary residual disease (71.1% vs. 55.3%; *p* = 0.079). No association was observed between ALNM TILs and axillary pCR (50% vs. 61.5%, *p* = 0.382) (Table 3).

**Figure 3:**
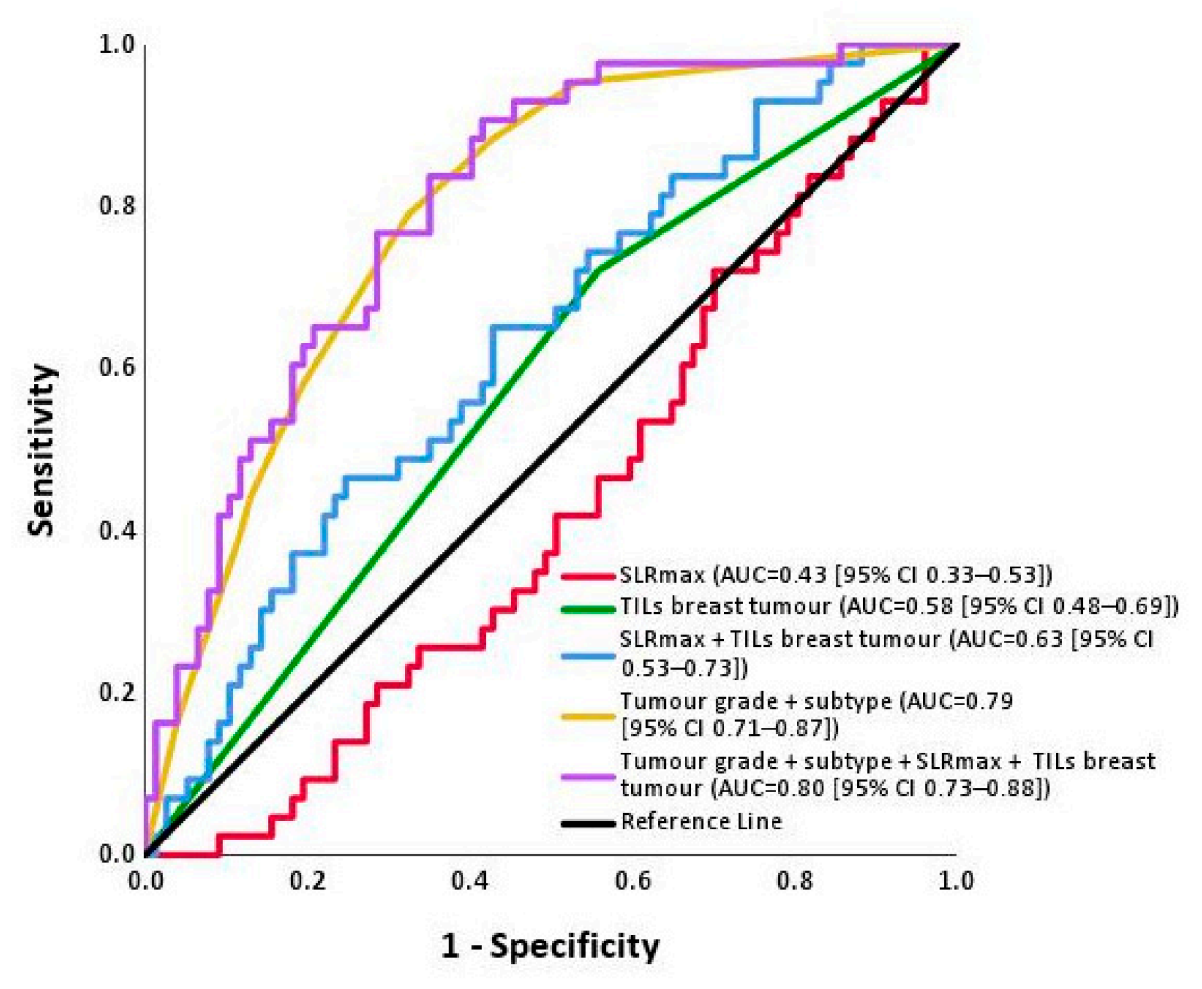
Receiver operating characteristic (ROC) curve analysis illustrating the discriminative ability of SLRmax, TILs breast tumour, SLRmax + TILs breast tumour, tumour grade + breast cancer subtype, and the multivariable model of Table 4 (Tumour grade + breast cancer subtype + SLRmax + TILs breast tumour) for axillary pCR. AUC, area under the curve; TILs, tumor-infiltrating lymphocytes; SLR, spleen-to-liver ratio; pCR, pathologic complete response.

Results of univariable and multivariable logistic regression analyses of factors associated with axillary pCR are presented in Table 4. Multivariable logistic regression combining tumour grade (grade 3 vs. 1–2; OR 2.19 [95% CI 0.87–5.55], *p* = 0.097), breast cancer subtype (Ref HR+/HER2−-; HR+/HER2+ OR 10.38 [95% CI 2.95–36.46], *p* < 0.001; HR−-/HER2+ OR 13.66 [95% CI 2.84–65.75], *p* = 0.001; TN OR 5.13 [95% CI 1.44–18.29], *p* = 0.012), SLRmax (OR 0.01 [95% CI 0.00–2.74], *p* = 0.111), and breast tumour TILs (OR 1.25 [95% CI 0.45–3.48], *p* = 0.666), resulted in an apparent AUC of 0.80 [95% CI 0.73–0.88]; Figure 3). In comparison, the multivariable model including only SLRmax and breast tumour TILs resulted in an apparent AUC of 0.63 [95% CI 0.53–0.73], while the multivariable model including only breast cancer subtype and tumour grade resulted in an apparent AUC of 0.79 [95% CI 0.71–0.87].

**Figure 4:**
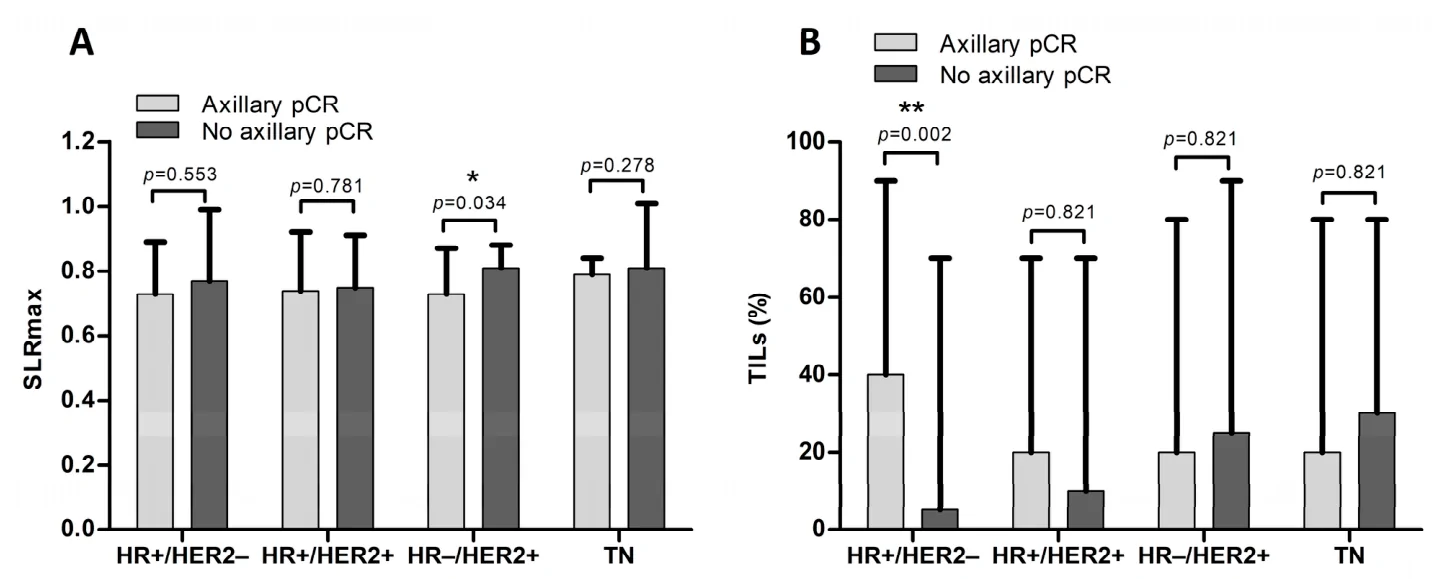
(**A**) Baseline maximum spleen-to-liver ratio (SLRmax) across patients with axillary pCR (light grey) and without axillary pCR (dark grey) according to breast cancer subtypes. (**B**) Percentage of pre-NAC TILs of the breast tumour (continuous) across patients with axillary pCR (light grey) and without axillary pCR (dark grey) according to breast cancer subtypes. pCR, pathologic complete response; SLR, spleen-to-liver ratio; HR, hormone receptor; HER2, human epidermal growth factor receptor 2; TN, triple negative; NAC, neoadjuvant chemo-(immuno)therapy; TILs, tumour-infiltrating lymphocytes. * *p* < 0.05; ** *p* < 0.01.

Figure 4 illustrates pre-NAC SLRmax and breast tumour TILs according to axillary pCR rates across breast cancer subtypes. Within the HR-negative/HER2-positive subtype, baseline SLRmax was significantly lower in patients achieving axillary pCR (*p* = 0.034; Figure 4A). The percentage of pre-NAC breast tumour TILs was significantly higher in patients achieving axillary pCR within the HR-positive/HER2-negative subtype (*p* = 0.002; Figure 4B). No significant differences in SLRmax and breast tumour TILs were observed between patients with and without axillary pCR in the other subtypes.

Table 5 summarises the association between [^18^F]FDG PET/CT-derived metabolic characteristics and breast tumour and ALNM TILs. For breast tumour [^18^F]FDG PET/CT-derived characteristics, SUVmax, SUVmean, MTV, TBR liver, TBR blood pool and TBR contralateral breast were all higher in patients with high breast tumour TILs; however, none of these differences reached statistical significance. Metabolic activity in ALNMs was significantly higher in patients with high breast tumour TILs (≥10%), reflected by an increased SUVmax (10.5 vs. 6.3, *p* = 0.024), SUVmean (6.4 vs. 3.7, *p* = 0.015), and NT-ratio (0.8 (0.2–5.0) vs. 0.7 (0.2–3.6), *p* = 0.013). At the systemic level, lower splenic SUVmean was observed in patients with high breast tumour TILs (≥10%) compared to those with low TIL levels (2.6 vs. 3.1, *p* = 0.035). No significant associations were observed between [^18^F]FDG PET/CT-derived metabolic characteristics and pre-NAC ALNM TIL levels.

## 4. Discussion

Patients with cN+ breast cancer who achieve axillary pCR after NAC may not benefit from any axillary surgery. Therefore, accurate non-invasive identification of patients achieving axillary pCR may allow omission of axillary surgery in the future. In this study, we explored the value of baseline [^18^F]FDG PET/CT-derived metabolic characteristics and TILs to predict axillary pCR in cN+ breast cancer patients treated with NAC by creating a multivariable model. The model combining [^18^F]FDG PET/CT-derived SLRmax and breast tumour TILs demonstrated moderate performance (apparent AUC 0.63 [95% CI 0.53–0.73]). A model including only the clinicopathological characteristics, breast cancer subtype and tumour grade, performed considerably better (apparent AUC 0.79 [95% CI 0.71–0.87], underscoring the importance of including tumour biology in response prediction. Adding SLRmax and breast tumour TILs to this clinicopathological model yielded a similar performance (apparent AUC 0.80 [95% CI 0.73–0.88]), so an incremental predictive value of these biomarkers could not be demonstrated in our cohort. Second, the association between [^18^F]FDG PET/CT-derived metabolic characteristics and TILs revealed higher ALNM metabolic activity and lower splenic metabolic activity in patients with high breast tumour TILs.

Regarding [^18^F]FDG PET/CT-derived metabolic characteristics, a trend towards higher breast tumour and ALNM [^18^F]FDG uptake was observed in patients achieving axillary pCR (SUVmax breast tumour 14.4 vs. 11.7, *p* = 0.096; SUVmax ALNM 9.6 vs. 8.3, *p* = 0.124). This is in contrast with previous studies, in which lower metabolic activity of the breast tumour and ALNMs was associated with achieving axillary pCR [9,10]. However, when stratified by breast cancer subtype, our data showed a shift towards lower breast tumour SUVmax values being associated with axillary pCR in all subtypes except TN breast cancer. This may reflect subtype-specific differences in tumour biology, where lower metabolic activity within one subtype may indicate a less aggressive tumour, associated with improved treatment response [9]. Regarding splenic metabolic activity on [^18^F]FDG PET/CT, a trend towards lower SUV values was observed in patients achieving axillary pCR. To our knowledge, no prior studies evaluated the association between splenic metabolic activity and pCR in breast cancer. However, multiple previous studies in breast cancer as well as in other cancer types have reported higher splenic metabolic values to be associated with worse prognosis [12,13,15,33,34]. Lower splenic metabolic activity may reflect a less active systemic immune response, or alternatively, a more effective tumour-directed immune response, contributing to improved treatment response and prognosis [15,20].

Regarding the tumour-associated immune response, reflected by TILs, this study showed that patients with high levels of breast tumour TILs (≥10%) more often achieved axillary pCR. This observation is in accordance with results of previous studies, which also described higher pCR rates and better disease-free and overall survival rates in patients with high levels of breast tumour TILs [18,35,36]. However, the independent predictive value of TILs may be limited in multivariable analyses of our study due to their association with breast cancer subtype and tumour grade. No association was observed between ALNM TILs and axillary pCR. To our knowledge, only a limited number of previous studies have evaluated immune cell infiltration in ALNMs, all focusing on specific immune cell subsets rather than standardised TIL assessment [37,38]. This may be due to the methodological challenges associated with measuring the level of ALNM TILs [35]. In the absence of tumour stroma, it is difficult to differentiate lymphocytes physiologically present in the ALNs from lymphocytes migrating to ALNMs as part of the immune response to attack the tumour cells [35,39]. Therefore, the reliability of ALNM TIL assessment remains questionable. Overall, these findings confirm the relevance of breast tumour TILs for the prediction of axillary pCR, whereas ALNM TILs, although not previously investigated, do not appear to have a predictive value for axillary pCR.

Concerning the relationship between [^18^F]FDG PET/CT-derived metabolic characteristics and TILs, our study demonstrated significantly higher metabolic activity in ALNMs among patients with high breast tumour TILs compared to those with low TILs. This aligns with Seban et al., who also reported a significant positive correlation between ALNM SUVmax and breast tumour TIL levels [21]. Most other previous studies focused on the correlation between breast tumour [^18^F]FDG uptake and breast tumour TIL levels, reporting a higher uptake with increasing TILs [19,22]. In our study, a trend was observed between breast tumour metabolic activity and TILs. This may be explained by differences in the usage of TIL cut-offs, nodal status and included breast cancer subtypes. Moreover, ALNMs represent key sites of immune activity, where TILs are being activated as part of the anti-tumour immune response and subsequently recruited into tumour tissue [40]. This immunological interaction may explain the stronger association between ALNM metabolic activity and breast tumour TILs; however, future studies are needed to confirm this. Regarding splenic metabolic activity, we observed a trend towards an inverse association with breast tumour TILs, which may suggest migration of systemic splenic immune cells to the local tumour microenvironment. This is in line with findings of Seban et al., who reported higher splenic glucose metabolism to be associated with a more immunosuppressive tumour microenvironment, characterised by lower breast tumour TILs [15]. In contrast, Tamam et al. reported a positive association between splenic metabolic activity and breast tumour TILs in stage 4 breast cancer, whereas in stage 2 disease, a weak inverse association was observed [20]. These differences may reflect variation in systemic immune activation across disease stages [20].

This study has several limitations. First, [^18^F]FDG PET/CT exams and CNBs were collected retrospectively. Although this enabled a centralised reassessment of both [^18^F]FDG PET and TIL measurements by a single radiologist and pathologist, interobserver variability was not assessed. Second, the absence of post-NAC [^18^F]FDG PET/CT imaging precluded the assessment of changes in quantitative PET parameters (e.g., SUV) in both the breast tumour and ALNMs as well as in non-target organs, such as the liver and spleen, which may provide additional information. Third, [^18^F]FDG PET/CT exams were collected from 13 different centres in the Netherlands. Because acquisition and reconstruction protocols differ between centres, we included only scans from EARL-accredited centres (nine centres) for this retrospective analysis. This selection may have introduced selection bias and limits the generalisability of our findings. Although EARL accreditation harmonises quantitative measurements across scanners, minor residual differences in scanner type, reconstruction protocol and acquisition parameters cannot be fully excluded. Fourth, detailed information on patients’ medical history was lacking. Therefore, patients with conditions that may have influenced immune function, such as chronic inflammation, autoimmune disorders, or those who used immunomodulatory medication, were not excluded from analysis, while these are known to potentially influence both splenic metabolic activity and levels of TILs [41,42]. Fifth, as no internal or external validation was performed and model calibration was not assessed, the reported AUCs reflect apparent performance in the development cohort. As a result, model performance may be overestimated, and generalisability to independent populations remains to be established. Sixth, variable selection was partly based on univariable significance, which may result in unstable models and overestimated effect sizes, particularly given the limited number of axillary pCR events (n = 46). Together with the absence of internal and external validation, this underlines that the model should be considered exploratory and hypothesis-generating.

Future studies should aim to further optimise model performance before omission of axillary surgery can be considered, as models would need to achieve high discriminative performance, in particular a very low false-negative rate, to avoid undertreatment and safely guide treatment de-escalation.

## 5. Conclusions

In conclusion, this study found that the model combining clinicopathological characteristics, splenic metabolic activity, and breast tumour TILs may be informative for the axillary response to NAC but did not perform better than clinicopathological characteristics alone. Increased metabolic activity of ALNMs was associated with high breast tumour TILs, while an inverse relationship with splenic metabolic activity was observed. Validation in independent cohorts is required before any clinical application can be considered, and future studies should evaluate whether sufficiently accurate prediction models can support safe treatment de-escalation.

## Figures and Tables

**Table 1:** Patient and tumour characteristics according to axillary pCR status.

Characteristic	Total (n = 135)	Ax pCR (n = 46)	No Ax pCR (n = 89)	*p*-Value
Age at diagnosis (years), mean ± SD	51.5 ± 11.6	52.2 ± 12.3	51.1 ± 11.3	0.635 ^a^
cT-status, n (%)				0.521 ^b^
cT1	14 (10.3)	4 (8.7)	10 (11.2)	
cT2	81 (60.0)	28 (60.9)	53 (59.6)	
cT3	35 (25.9)	11 (23.9)	24 (27.0)	
cT4	4 (3.0)	3 (6.5)	1 (1.1)	
Occult	1 (0.7)	0 (0.0)	1 (1.1)	
cN-status, n (%)				0.293 ^b^
cN1	96 (71.1)	29 (63.0)	67 (75.3)	
cN2	29 (21.5)	12 (26.1)	17 (19.1)	
cN3b	10 (7.4)	5 (10.9)	5 (5.6)	
Oestrogen receptor, n (%)				**<0.001** ^b^
Negative	53 (39.3)	28 (60.9)	25 (28.1)	
Positive	82 (60.7)	18 (39.1)	64 (71.9)	
Progesterone receptor, n (%)				**0.003** ^b^
Negative	64 (47.4)	30 (65.2)	34 (38.2)	
Positive	71 (52.6)	16 (34.8)	55 (61.8)	
HER2 receptor, n (%)				**<0.001** ^b^
Negative	93 (68.9)	19 (41.3)	74 (83.1)	
Positive	42 (31.1)	27 (58.7)	15 (16.9)	
Breast cancer subtype, n (%)				**<0.001** ^b^
HR+/HER2−	58 (43.0)	5 (10.9)	53 (59.6)	
HR+/HER2+	28 (20.7)	17 (37.0)	11 (12.4)	
HR−/HER2+	14 (10.4)	10 (21.7)	4 (4.5)	
HR−/HER2 − (TN)	35 (25.9)	14 (30.4)	21 (23.6)	
Tumour grade (mBR), n (%)				**0.005** ^b^
Grade 1	9 (6.7)	1 (2.2)	8 (9.0)	
Grade 2	59 (43.7)	14 (30.4)	45 (50.6)	
Grade 3	57 (42.2)	29 (63.0)	28 (31.5)	
Missing	10 (7.4)	2 (4.3)	8 (9.0)	
Histology type, n (%)				0.499 ^b^
Invasive NST	130 (96.3)	45 (97.8)	85 (95.5)	
ILC	5 (3.7)	1 (2.2)	4 (4.5)	

Bold values indicate statistical significance (*p* < 0.05); ^a^ Unpaired t-test; ^b^ Chi-square test. pCR, pathologic complete response; cT-status, clinical tumour status; cN-status, clinical nodal status; HER2, human epidermal growth factor receptor 2; HR, hormone receptor; TN, triple negative; mBR, modified Bloom–Richardson grade; NST, no special type; ILC, invasive lobular carcinoma.

**Table 2:** [^18^F]FDG PET/CT imaging characteristics according to axillary pCR status.

Characteristic	n	Total (n = 135) Median (Range)	n	Axillary pCR (n = 46) Median (Range)	n	No Axillary pCR Median (Range)	*p*-Value
Serum glucose (mmol/L)	130	5.2 (3.8–7.2)	44	5.3 (3.9–6.6)	86	5.2 (3.8–7.2)	0.710
[^18^F]FDG dose (MBq)	133	166.0 (100.0–400.0)	45	174.7 (100.0–400.0)	88	163.4 (100.0–400.0)	0.471
Breast tumour							
SUVmax	135	13.0 (1.2–51.7)	46	14.4 (1.4–46.4)	89	11.7 (1.2–51.7)	0.096
SUVmean	134	7.9 (0.8–34.1)	46	9.1 (1.0–29.2)	88	7.2 (0.8–34.1)	0.117
MTV (cm^3^)	135	4.0 (0.6–31.6)	46	4.0 (0.6–30.0)	89	4.0 (0.7–31.6)	0.856
TBR liver							
SUVmax	135	2.6 (0.2–9.3)	46	3.4 (0.2–9.3)	89	2.2 (0.3–7.2)	0.055
SUVmean	134	2.1 (0.2–6.7)	46	2.7 (0.2–6.7)	88	1.9 (0.3–6.3)	**0.042**
TBR mediastinal blood pool							
SUVmax	135	3.5 (0.3–11.2)	46	4.9 (0.3–11.2)	89	3.3 (0.5–9.2)	0.072
SUVmean	134	2.9 (0.3–9.2)	46	4.0 (0.3–8.9)	88	2.6 (0.4–9.2)	0.085
TBR contralateral breast							
SUVmax	135	5.6 (0.9–42.9)	46	5.9 (0.9–42.9)	89	5.2 (1.2–19.9)	0.122
SUVmean	134	5.2 (0.7–47.5)	46	5.9 (0.7–47.5)	88	4.7 (1.1–22.9)	0.179
Most hypermetabolic ALN							
SUVmax	135	8.7 (1.2–43.4)	46	9.6 (2.7–34.3)	89	8.3 (1.2–43.4)	0.124
SUVmean	135	5.3 (0.8–29.2)	46	6.0 (1.8–19.9)	89	5.1 (0.8–29.2)	0.119
No. suspected ALNs	135	3 (0–13)	46	3 (1–13)	89	3 (0–11)	0.779
NT-ratio							
SUVmax	135	0.8 (0.2–5.0)	46	0.7 (0.2–4.9)	89	0.8 (0.2–5.0)	0.636
SUVmean	134	0.8 (0.2–4.8)	46	0.8 (0.2–4.8)	88	0.8 (0.2–2.6)	0.488
Spleen							
SUVmax	135	3.7 (1.8–10.5)	46	3.4 (1.8–10.5)	89	3.7 (1.9–10.5)	0.415
SUVmean	135	2.8 (1.4–7.3)	46	2.7 (1.4–7.3)	89	2.8 (1.8–7.0)	0.309
SLR							
SUVmax	135	0.8 (0.6–1.0)	46	0.7 (0.6–0.9)	89	0.8 (0.6–1.0)	0.169
SUVmean	135	0.8 (0.6–1.1)	46	0.8 (0.6–0.9)	89	(0.8 (0.6–1.1)	0.531

Bold values indicate statistical significance (*p* < 0.05); pCR, pathologic complete response; MBq, megabecquerel; MTV, metabolic tumour volume; SUV, standardised uptake value; ALN, axillary lymph node; TBR, tumour-to-background ratio; NT-ratio, nodal-to-tumour ratio; SLR, spleen-to-liver ratio.

**Table 3:** Association between breast tumour and ALNM TIL levels and axillary pCR status.

Characteristic	Total	Axillary pCR	No Axillary pCR	*p*-Value
Breast tumour TILs, n (%)				0.079
Low (<10)	51 (39.2)	13 (28.9)	38 (44.7)	
High (≥10)	79 (60.8)	32 (71.1)	47 (55.3)	
ALNM TILs, n (%)				0.382
Low (<10)	26 (42.6)	11 (50.0)	15 (38.5)	
High (≥10)	35 (57.4)	11 (50.0)	24 (61.5)	

pCR, pathologic complete response; TILs, tumour-infiltrating lymphocytes.

**Table 4:** Univariable and multivariable logistic regression analysis of characteristics associated with axillary pCR.

		Univariable			Multivariable		
Characteristic	n	OR	95% CI	*p*-Value	OR	95% CI	*p*-Value
Clinicopathologic characteristics							
Age							
≤50	59	Ref					
>50	73	1.02	0.49–2.10	0.967			
cT-status							
cT1-2	95	Ref					
cT3-4	39	1.10	0.51–2.41	0.806			
cN-status							
cN1	96	Ref					
cN2-3b	39	1.79	0.83–3.85	0.139			
Tumour grade (mBR)							
Grade 1–2	68	Ref			Ref		
Grade 3	57	3.66	1.69–7.93	**0.001**	2.19	0.87–5.55	0.097
Breast cancer subtype							
HR+/HER2−	58	Ref			Ref		
HR+/HER2+	28	16.38	4.98–53.85	**<0.001**	10.38	2.95–36.46	**<0.001**
HR−/HER2+	14	26.50	6.04–116.21	**<0.001**	13.66	2.84–65.75	**0.001**
TN	35	7.07	2.26–22.08	**<0.001**	5.13	1.44–18.29	**0.012**
[^18^F]FDG PET/CT characteristics							
Breast tumour							
SUVmax	135	1.03	0.99–1.07	0.143			
SUVmean	134	1.04	0.98–1.11	0.194			
MTV (cm^3^)	135	1.01	0.95–1.07	0.782			
TBR liver							
SUVmax	135	1.28	1.02–1.59	**0.032**			
SUVmean	134	1.34	1.03–1.74	**0.032**			
TBR mediastinal blood pool							
SUVmax	135	1.17	1.00–1.37	**0.047**			
SUVmean	134	1.17	0.97–1.42	0.100			
TBR contralat. breast							
SUVmax	135	1.07	1.01–1.15	**0.034**			
SUVmean	134	1.06	1.00–1.12	0.059			
Most hypermetabolic ALN							
SUVmax	135	1.02	0.98–1.06	0.397			
SUVmean	135	1.03	0.96–1.09	0.434			
No. suspected ALNs							
1–3	87	Ref					
≥4	48	0.82	0.39–1.74	0.607			
NT-ratio							
SUVmax	135	1.23	0.79–1.93	0.358			
SUVmean	134	1.50	0.88–2.56	0.137			
Spleen							
SUVmax	135	1.01	0.81–1.26	0.911			
SUVmean	135	0.96	0.69–1.34	0.826			
SLR							
SUVmax	135	0.05	0.00–3.03	0.150	0.01	0.00–2.74	0.111
SUVmean	135	0.12	0.00–7.32	0.310			
TILs characteristics							
TILs breast tumour							
<10	51	Ref			Ref		
≥10	79	1.99	0.92–4.31	0.081	1.25	0.45–3.48	0.666
TILs ALNMs							
<10	26	Ref					
≥10	35	0.63	0.22–1.80	0.383			

Bold values indicate statistical significance (*p* < 0.05). OR, odds ratio; CI, confidence interval; cT-status, clinical tumour status; cN-status, clinical nodal status; mBR, modified Bloom–Richardson grade; HR, hormone receptor; HER2, human epidermal growth factor receptor 2; TN, triple negative; TILs, tumour-infiltrating lymphocytes; ALNM, axillary lymph node metastasis; SUV, standardised uptake value; MTV, metabolic tumour volume; TBR, tumour-to-background ratio; ALN, axillary lymph node; NT-ratio, nodal-to-tumour ratio; SLR, spleen-to-liver ratio.

**Table 5:** [^18^F]FDG PET/CT imaging characteristics according to breast tumour and axillary TIL levels.

	Breast Tumour TILs					ALNM TILs				
Characteristic	n	Low Breast Tumour TILs (<10%)	n	High Breast Tumour TILs (≥10%)	*p*-Value	n	Low ALNM TILs (<10%)	n	High ALNM TILs (≥10%)	*p*-Value
Serum glucose (mmol/L)	49	5.2 (3.9–7.2)	76	5.3 (3.8–7.0)	0.300	25	5.3 (4.4–7.2)	34	5.2 (4.4–6.8)	0.484
[^18^F]FDG dose (MBq)	51	163.9 (100.0–400.0)	78	170.4 (100.0–400.0)	0.505	26	163.4 (100.0–297.0)	35	173.0 (102.1–400.0)	0.484
Breast tumour										
SUVmax	51	12.4 (1.4–46.4)	79	13.1 (1.6–51.7)	0.788	26	11.0 (1.4–29.8)	35	11.7 (1.2–40.7)	0.988
SUVmean	51	7.4 (1.0–29.2)	78	8.2 (1.0–34.1)	0.649	26	6.9 (1.0–17.3)	35	6.8 (0.8–24.2)	0.861
MTV (cm^3^)	51	3.7 (0.6–30.0)	79	4.2 (1.0–31.6)	0.141	26	5.1 (1.1–28.4)	35	5.0 (1.0–30.0)	0.579
TBR liver										
SUVmax	51	2.2 (0.2–5.4)	79	2.8 (0.5–9.3)	0.110	26	2.8 (0.2–6.5)	35	2.2 (0.3–9.3)	0.850
SUVmean	51	1.9 (0.2–4.9)	78	2.3 (0.4–6.7)	0.122	26	2.2 (0.2–5.1)	35	1.7 (0.3–6.7)	0.748
TBR blood pool										
SUVmax	51	3.2 (0.3–7.7)	79	4.2 (0.6–11.2)	0.159	26	3.8 (0.3–8.2)	35	3.3 (0.5–11.2)	0.610
SUVmean	51	2.5 (0.3–6.3)	78	3.3 (0.5–8.9)	0.138	26	2.8 (0.3–6.3)	35	2.3 (0.4–9.2)	0.748
TBR contralateral breast										
SUVmax	51	5.5 (1.0–18.4)	79	5.7 (0.9–42.9)	0.318	26	5.2 (1.0–42.9)	35	5.6 (0.9–24.6)	0.672
SUVmean	51	5.0 (0.9–22.9)	78	5.3 (0.7–47.5)	0.413	26	4.6 (0.9–47.5)	35	5.1 (0.7–23.5)	0.988
Most hypermetabolic ALN										
SUVmax	51	6.3 (1.2–34.3)	79	10.5 (2.6–43.4)	**0.024**	26	6.5 (1.6–34.7)	35	7.9 (2.7–31.2)	0.390
SUVmean	51	3.7 (0.8–21.1)	79	6.4 (1.6–29.2)	**0.015**	26	4.1 (1.2–21.1)	35	4.2 (1.7–19.3)	0.453
No. suspected ALNs	51	3.0 (0–6)	79	3 (1–13)	0.170	26	3 (1–11)	35	3 (1–9)	0.710
NT-ratio										
SUVmax	51	0.7 (0.2–3.6)	79	0.8 (0.2–5.0)	**0.013**	26	0.7 (0.2–3.6)	35	0.7 (0.2–4.9)	0.448
SUVmean	51	0.7 (0.2–2.8)	78	0.8 (0.2–4.8)	**0.015**	26	0.8 (0.2 2.8)	35	0.7 (0.2–4.8)	0.705
Spleen										
SUVmax	51	3.9 (1.9–7.4)	79	3.4 (1.8–10.5)	0.120	26	3.3 (1.9–5.7)	35	3.3 (2.0–10.5)	0.878
SUVmean	51	3.1 (1.9–5.6)	79	2.6 (1.4–7.3)	**0.035**	30	2.6 (1.8–4.4)	31	2.5 (1.4–7.0)	0.942
SLR										
SUVmax	51	0.7 (0.6–1.0)	79	0.8 (0.6–1.0)	**0.043**	26	0.7 (0.6–0.9)	35	0.8 (0.6–0.9)	0.737
SUVmean	51	0.8 (0.6–1.1)	79	0.8 (0.6–1.0)	0.466	26	0.8 (0.6–1.0)	35	0.8 (0.6–1.1)	0.274

Bold values indicate statistical significance (*p* < 0.05). TILs, tumour-infiltrating lymphocytes; MBq, megabecquerel; SUV, standardised uptake value; MTV, metabolic tumour volume; TBR, tumour-to-background ratio; ALN, axillary lymph node; NT-ratio, nodal-to-tumour ratio; SLR, spleen-to-liver ratio.

## Data Availability

Data and code for the final analyses are available from the corresponding author upon reasonable request.

## Supplementary Materials

The following supporting information can be downloaded at: https://www.mdpi.com/article/10.3390/cancers18203302/s1, Table S1: [^18^F]FDG PET/CT imaging characteristics according to axillary pCR status across breast cancer subtypes; Collaborators REFINE study group.
