# Supplementary material for: [18F]FDG PET/CT-Derived Metabolic Characteristics and Tumour-Infiltrating Lymphocytes for Predicting Axillary Response to Neoadjuvant Chemo-(Immuno)Therapy in Clinically Node-Positive Breast Cancer
Source: Cancers (Basel). 2026 Oct 14;18(20):3302. doi: 10.3390/cancers18203302 (PMC13702557; doi:10.3390/cancers18203302)
Supplement: Supplementary file 1 [file cancers-18-03302-s001.zip › cancers-4573195-supplementary-author proofread.pdf]

Table S1. [^{18}F]FDG PET/CT imaging characteristics according to axillary pCR status across breast cancer subtypes

**Characteristic**	**HR+ HER2–**					**HR+ HER2+**					**HR–- HER2+**					**TN**				
	**n**	**Ax pCR**	**n**	**Ax RD**	**p-value**	**n**	**Ax pCR**	**n**	**Ax RD**	**p-value**	**n**	**Ax pCR**	**n**	**Ax RD**	**p-value**	**n**	**Ax pCR**	**n**	**Ax RD**	**p-value**
Serum glucose (mmol/L)	5	5.1 (4.5-6.0)	51	5.3 (3.8-6.7)	0.676	16	5.3 (4.8-6.0)	11	5.2 (4.5-7.2)	0.680	10	5.3 (3.9-6.6)	4	5.2 (4.4-6.5)	0.776	13	5.6 (4.6-6.2)	20	5.2 (3.9-7.0)	0.624
[^{18}F]FDG dose (MBq)	5	150 (100-284)	52	158.3 (105.5-400)	0.880	16	157 (100-400)	11	163.4 (129.6-400)	0.481	10	233.3 (124.1-282)	4	226.5 (186.9-279)	0.839	14	170.8 (102.1-297)	21	193 (100-337)	0.495
Breast tumour																				
SUVmax	5	8.2 (1.6-11.8)	53	10.0 (1.2-32.8)	0.253	17	14.3 (3.8-46.4)	11	15.2 (6.3-24.1)	0.890	10	15.4 (1.4-30.3)	4	21.2 (8.7-37.0)	0.671	14	18.0 (2.6-29.8)	21	15.1 (3.5-51.7)	0.495
SUVmean	5	4.8 (1.0-7.7)	53	6.2 (0.8-20.4)	0.253	17	9.0 (2.2-29.2)	11	8.8 (3.6-16.2)	0.926	10	9.2 (1.0-19.1)	4	12.2 (5.1-23.8)	0.733	14	10.7 (1.6-17.3)	20	9.8 (2.7-34.1)	0.616
MTV (cm^3)	5	6.0 (3.8-10.6)	53	3.9 (0.7-23.3)	0.131	17	4.2 (0.7-12.5)	11	5.0 (1.6-26.2)	0.378	10	3.0 (0.9-18.2)	4	5.4 (1.2-8.2)	0.635	14	3.5 (0.6-30.0)	21	3.9 (1.1-31.6)	0.855
TBR liver																				
SUVmax	5	2.3 (0.5-5.1)	53	2.0 (0.3-7.2)	0.872	17	3.3 (0.9-5.2)	11	2.9 (1.3-5.9)	0.963	10	2.2 (0.2-9.3)	4	3.0 (1.7-5.3)	0.454	14	4.8 (0.5-6.5)	21	2.6 (0.9-6.1)	0.061
SUVmean	5	2.0 (0.4-4.3)	53	1.5 (0.3-6.3)	0.830	17	2.6 (0.7-4.9)	11	2.4 (1.1-5.2)	0.926	10	2.2 (0.2-6.7)	4	2.4 (1.4-4.9)	0.454	14	3.7 (0.4-5.5)	20	2.5 (0.9-4.9)	0.077
TBR blood pool																				
SUVmax	5	3.1 (0.7-6.7)	53	2.9 (0.5-8.7)	0.936	17	5.5 (1.1-7.7)	11	4.2 (1.6-7.6)	0.677	10	3.5 (0.3-11.2)	4	5.1 (2.4-9.2)	0.454	14	6.3 (0.7-10.1)	21	4.2 (1.2-9.0)	0.186
SUVmean	5	2.3 (0.6-6.1)	53	2.3 (0.4-9.2)	0.957	17	3.6 (0.8-6.1)	11	3.3 (1.3-7.0)	0.711	10	2.9 (0.3-8.9)	4	3.9 (1.7-7.5)	0.539	14	4.5 (0.5-7.6)	20	3.4 (1.2-7.0)	0.180
TBR contralateral breast																				
SUVmax	5	4.1 (0.9-11.9)	53	4.0 (1.2-18.3)	0.914	17	5.8 (2.0-18.4)	11	6.3 (3.1-12.9)	0.926	10	5.5 (1.0-24.6)	4	12.0 (5.4-19.9)	0.304	14	9.7 (1.7-42.9)	21	6.7 (1.2-19.8)	0.145
SUVmean	5	3.2 (0.7-12.8)	53	3.8 (1.1-22.4)	0.747	17	5.8 (1.4-22.9)	11	5.0 (2.8-11.3)	1.000	10	5.2 (0.9-23.5)	4	12.2 (4.7-22.9)	0.454	14	9.0 (1.5-47.5)	20	5.7 (2.3-21.6)	0.129
Most hypermetabolic ALN																				
SUVmax	5	7.9 (4.0-16.5)	53	5.8 (1.2-34.1)	0.535	17	9.8 (2.7-29.9)	11	8.1 (2.6-25.7)	0.746	10	9.3 (5.0-30.5)	4	22.3 (3.6-34.7)	0.454	14	11.2 (3.0-34.3)	21	14.2 (2.6-43.4)	0.678
SUVmean	5	4.9 (2.6-10.3)	53	3.6 (0.8-21.1)	0.433	17	6.4 (1.9-19.9)	11	4.4 (1.6-16.1)	0.611	10	5.6 (2.9-18.0)	4	13.8 (2.2-20.8)	0.454	14	7.0 (1.8-19.5)	21	8.6 (1.7-29.2)	0.606
No. susp ALNs	5	3 (2-7)	53	2 (0-6)	0.180	17	3 (1-13-)	11	3 (1-5)	0.711	10	3 (1-6)	4	3.5 (3-11)	0.304	14	1.5 (1-11)	21	3 (1-9)	0.127
NT-ratio																				
SUVmax	5	1.3 (0.5-4.9)	53	0.8 (0.2-3.1)	0.154	17	0.7 (0.3-1.4)	11	0.7 (0.4-1.4)	1.000	10	0.7 (0.3-3.6)	4	0.9 (0.4-1.2)	0.945	14	0.8 (0.2-2.8)	21	0.8 (0.3-5.0)	0.516
SUVmean	5	1.2 (0.5-4.8)	53	0.8 (0.2-2.6)	0.154	17	0.8 (0.3-1.5)	11	0.7 (0.3-1.5)	0.517	10	0.8 (0.3-2.8)	4	0.9 (0.4-1.2)	1.000	14	0.8 (0.2-3.1)	20	0.8 (0.3-2.0)	0.545
Spleen																				
SUVmax	5	2.1 (2.0-3.1)	53	3.7 (1.9-7.2)	**0.001**	17	3.3 (1.8-10.5)	11	3.3 (2.3-10.5)	0.611	10	4.5 (2.5-8.7)	4	5.7 (4.0-6.1)	0.733	14	3.3 (2.0-5.1)	21	3.7 (2.4-7.6)	0.127
SUVmean	5	1.7 (1.4-2.2)	53	2.8 (1.9-5.3)	**<0.001**	17	2.8 (1.6-7.3)	11	2.5 (2.0-7.0)	0.677	10	3.4 (2.1-6.3)	4	4.3 (3.0-4.4)	0.454	14	2.4 (1.6-3.6)	21	2.8 (1.8-5.2)	0.083
SLR																				
SUVmax	5	0.7 (0.6-0.9)	53	0.8 (0.6-1.0)	0.553	17	0.7 (0.7-0.9)	11	0.8 (0.7-0.9)	0.781	10	0.7 (0.6-0.9)	4	0.8 (0.8-0.9)	**0.036**	14	0.8 (0.7-0.8)	21	0.8 (0.7-1.0)	0.278
SUVmean	5	0.7 (0.6-0.9)	53	0.8 (0.6-1.1)	0.154	17	0.8 (0.7-0.9)	11	0.8 (0.7-1.0)	0.208	10	0.8 (0.6-0.9)	4	0.9 (0.8-0.9)	0.106	14	0.8 (0.7-0.9)	21	0.8 (0.7-1.1)	0.454

Bold values indicate statistical significance ($p<0.05$); HR, hormone receptor; HER2, human epidermal growth factor receptor 2; TN, triple negative; pCR, pathologic complete response; RD, residual disease; MBq, megabecquerel; SUV, standardized uptake value; MTV, metabolic tumour volume; TBR, tumour-to-background ratio; ALN, axillary lymph node; NT-ratio, nodal-to-tumour ratio; SLR, spleen-to-liver ratio.
