# Supplementary material for: [18F]FDG PET/CT-Derived Metabolic Characteristics and Tumour-Infiltrating Lymphocytes for Predicting Axillary Response to Neoadjuvant Chemo-(Immuno)Therapy in Clinically Node-Positive Breast Cancer
Source: Cancers (Basel). 2026 Oct 14;18(20):3302. doi: 10.3390/cancers18203302 (PMC13702557; doi:10.3390/cancers18203302)
Supplement: Supplementary file 1 [file cancers-18-03302-s001.zip › cancers-4573195-supplementary-Collaborators REFINE study group.pdf]

Collaborators REFINE study group

L. de Beer (Martini Hospital, Groningen, the Netherlands); E.G. Boerma (Zuyderland Medical Center, Sittard-Geleen, the Netherlands); M. Boskamp (Wilhelmina Hospital, Assen, the Netherlands); E.M.J. Brouwers-Kuyper (Albert Schweitzer Hospital, Dordrecht, the Netherlands); C.M.E. Contant (Maasstad Hospital, Rotterdam, the Netherlands); A.W.F. du Mée (Amphia Hospital, Breda, the Netherlands); H.J. Heijmans (Hospital Group Twente, Hengelo, the Netherlands); S. Ho-Han (Albert Schweitzer Hospital, Dordrecht, the Netherlands); F. Hulsebosch (Franciscus Gasthuis & Vlietland, Schiedam, the Netherlands); A. Jager (Erasmus MC Cancer Institute, University Medical Center Rotterdam, Rotterdam, the Netherlands); B.L.R. Kam (Diakonessenhuis, Utrecht, the Netherlands); W. Kelder (Martini Hospital, Groningen, the Netherlands); T.M.A.L. Klem (Franciscus Gasthuis & Vlietland, Schiedam, the Netherlands); M.B.I. Lobbes (Zuyderland Medical Center, Sittard-Geleen, the Netherlands); M.B.E. Menke- Pluijmers (Albert Schweitzer Hospital, Dordrecht, the Netherlands); C. de Monyé (Erasmus MC Cancer Institute, University Medical Center Rotterdam, Rotterdam, the Netherlands); P. Sars (Bravis Hospital, Roosendaal, the Netherlands); L.H.M. Smit (Treant Zorggroep Hospital, Hoogeveen, the Netherlands); E. van Haaren (Zuyderland Medical Center, Sittard-Geleen, the Netherlands); D. van Klaveren (Erasmus MC Cancer Institute, University Medical Center Rotterdam, Rotterdam, the Netherlands); J. Veltman (Hospital Group Twente, Hengelo, the Netherlands); C. Verhoef (Erasmus MC Cancer Institute, University Medical Center Rotterdam, Rotterdam, the Netherlands); W.J. Vles (Ikazia Hospital, Rotterdam, the Netherlands).
